# Taurine Attenuates Carcinogenicity in Ulcerative Colitis-Colorectal Cancer Mouse Model

**DOI:** 10.1155/2020/7935917

**Published:** 2020-05-20

**Authors:** Guifeng Wang, Ning Ma, Feng He, Shosuke Kawanishi, Hatasu Kobayashi, Shinji Oikawa, Mariko Murata

**Affiliations:** ^1^Department of Environmental and Molecular Medicine, Mie University Graduate School of Medicine, Tsu, Mie 514-8507, Japan; ^2^Sakuranomori Shiroko Home, Social Service Elderly Facilities, Suzuka University of Medical Science, Suzuka, Mie 513-0816, Japan; ^3^Graduate School of Health Science, Suzuka University of Medical Science, Suzuka, Mie 513-8670, Japan; ^4^Institute of Traditional Chinese Medicine, Suzuka University of Medical Science, Suzuka, Mie 510-0226, Japan; ^5^Graduate School of Pharmaceutical Sciences, Suzuka University of Medical Science, Suzuka, Mie 513-8670, Japan

## Abstract

Taurine (2-aminoethane-sulfonic acid) is a type of amino acids and has numerous physiological and therapeutic functions, including anti-inflammation. However, there are few studies on the anticancer action of taurine. Our previous studies have demonstrated that taurine exhibits an apoptosis-inducing effect on human nasopharyngeal carcinoma cells *in vitro*. In this study, we have investigated whether taurine has an anticancer effect, using azoxymethane (AOM)/sulfate sodium (DSS)- induced mouse model for colon carcinogenesis. All mice, except those in control group, received a single intraperitoneal injection of AOM and DSS in the drinking water for 7 days twice, with 1-week interval. After the first DSS treatment, mice were given distilled water (model group) or taurine in the drinking water (taurine group) ad libitum. No tumor was observed in the control group. Taurine significantly suppressed AOM+DSS-induced tumor formation. Histopathological examination revealed AOM/DSS treatment induced colon cancer in all mice (8/8, 100%), and taurine significantly inhibited the progression of colon cancer (4/9, 44.4%). Taurine significantly attenuated cell proliferation in cancer tissues detected by Ki-67 staining. Taurine significantly increased the levels of an apoptosis marker cleaved caspase-9 and tumor suppressor protein PTEN. This is the first study that demonstrated that taurine significantly reduced carcinogenicity *in vivo* using AOM/DSS-induced colon cancer mouse model.

## 1. Introduction

Taurine (2-aminoethane-sulfonic acid) is a special amino acid containing sulfonate group and lacking carboxyl group and is found in high concentrations in many cells. Humans can endogenously synthesize taurine, but primarily depend on their diet for taurine, mostly found in seafood [1]. Therefore, it is considered a conditionally essential nutrient. Taurine has numerous physiological functions, including bile salt conjugation, osmoregulation, membrane stabilization, calcium modulation, antioxidation, and anti-inflammation [2–4]. Taurine has different biological effects in various systems or organs, such as the cardiovascular system, skeletal muscle, retina, liver, kidney, and nervous system [3, 5, 6]. Taurine is used in the treatment of congestive heart failure, liver disease [7], and recently, for the suppression of stroke-like seizures in mitochondrial encephalomyopathy, lactic acidosis, and stroke-like seizures (MELAS) syndrome [8]. Taurine is also used as an ingredient of dietary supplements for energy drink ingested prior to exercise and revitalizing beverage for recovery from fatigue. Although many useful effects of taurine intake are reported, there are few studies about the anticancer action of taurine. We proposed the mechanism for crosstalk between DNA damage and inflammation in the multiple steps of carcinogenesis [9]. Our previous studies have demonstrated that taurine exhibits an apoptosis-inducing effect on human nasopharyngeal carcinoma cells *in vitro* [10, 11]. Suzuki et al. [12] demonstrated that azoxymethane (AOM) and subsequent severe inflammation induced by sulfate sodium (DSS) resulted in a high incidence of colonic epithelial malignancy, which is a useful mouse model for inflammation-related carcinogenesis. The proposed mechanism may raise the possibility of the cancer prevention by taurine because of its anti-inflammatory activity. In this study, we investigated whether taurine has an anticancer effect, using AOM/DSS-induced mouse model for colorectal cancer.

## 2. Materials and Methods

### 2.1. Animals and Chemicals

In this study, 4-week-old male C57BL/6J mice were purchased from Japan SLC Inc. (Hamamatsu, Japan). All protocols for animal studies were approved by the committee of animal center of Mie University, Mie, Japan (approval no. 26-19-sai2-hen1). They were acclimated for 1 week with tap water and a pelleted diet, ad libitum, before the start of the experimentation. They were housed under controlled conditions of humidity (50 ± 10%), light (12/12 h light/dark cycle), and temperature (22 ± 2°C). A colonic carcinogen AOM and taurine (>99%) were purchased from Sigma Chemical Co. (St. Louis, MO). DSS with a molecular weight of 40,000 was purchased from ICN Biomedicals, Inc. (Aurora, OH).

### 2.2. Experimental Procedure

**Figure 1:**
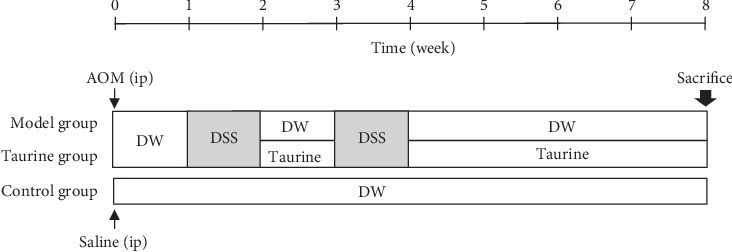
Experimental protocol.

Figure 1 shows the experimental protocol. All mice for AOM-DSS model received a single intraperitoneal injection (ip) of AOM at a dose level of 10 mg/kg body weight. One week and 3 weeks after the AOM injection, animals were exposed to 1.0% DSS (*W*/*V*) in the drinking water for 7 days twice, with one-week interval. After the first DSS treatment, the mice were randomly divided into two groups (*n* = 9, each) for DW and 0.5% (*W*/*V*) taurine in drinking water (model group and taurine group, respectively), ad libitum. The mice for control group (*n* = 3) were intraperitoneally injected saline and given distilled water. Body weight and stool status were check twice a week after DSS treatment. Then, they were then sacrificed by ether overdose at week 8. At autopsy, their large bowel was flushed with saline, and excised. The large bowel (from the ileocecal junction to the anal verge) was measured, cut open longitudinally along the main axis, and then washed with saline. Tumor lesions were counting micropathologically, by two investigators.

### 2.3. Fecal Blood Score

For scoring fecal blood status, the presence or absence of fecal blood was indicated as follows: 0 = negative hemoccult test, 1 = positive hemoccult test, and 2 = gross bleeding. Fecal occult blood of mice was detected by using a forensic luminol reaction kit (Luminol Reaction Experiment Kit, Wako Pure Chemical, Osaka, Japan), according to the instruction of the company and a study of Park and Tsunoda [13] in which they presented a simple protocol to detect fecal occult blood in mice, using this kit.

### 2.4. Histopathological and Immunohistochemical Studies

Colon tissue samples were fixed with 4% formaldehyde in phosphate buffered saline (PBS) for one day. Following dehydration and paraffin infiltration, the tumors were embedded in paraffin blocks and then sectioned to 5 *μ*m thickness using Leica Microsystems (Wetzlar, Germany) by routine procedures. Histopathological appearance of mouse tumors was evaluated by staining with hematoxylin and eosin (H&E) staining. Benign and malignant lesions were histopathologically distinguished using H&E staining samples by two investigators.

For immunohistochemistry (IHC) analysis, the paraffin-embedded mouse tumor sections were deparaffinized in xylene and series of alcohol. After the retrieval of heat-induced epitopes and blocking with 1% skim milk, sections were incubated overnight with primary antibodies (phosphatase and tensin homolog deleted on chromosome 10 (PTEN), Cell Signaling Technology, Inc., Danvers, MA #9188, 1: 400), Ki-67 (Proteintech Group Inc., Chicago, IL, 27309-1-AP, 1: 10,000), followed by incubation with biotinylated secondary antibodies (Vector Laboratories Burlingame, California, CA) for 2 h. The immunoreaction was visualized by a peroxidase stain DAB kit (Nacalai Tesque Inc., Kyoto, Japan). Nuclear counterstaining for PTEN staining samples was performed with hematoxylin, and tissues were observed and photographed under microscope (BX51, Olympus, Tokyo, Japan). The semiquantitative analysis of staining intensity was graded by an IHC score between 0 and 4 by two investigators as follows: no staining (0), weak staining (1+), moderate staining (2+), strong staining (3+), and very strong staining (4+) in all IHC studies.

### 2.5. Western Blot Analysis

A part of colon tissue samples (model group, *n* = 4; taurine group, *n* = 4; control group, *n* = 3) were immediately stored at -80°C until use. They were homogenized and lysed using RIPA buffer (Cell Signaling Technology Inc.) supplemented with phenylmethylsulfonyl fluoride (PMSF, Nacalai Tesque Inc.). Equal amounts of protein were separated by SDS-PAGE and transferred to polyvinylidene fluoride (PVDF) membranes (0.45 *μ*m, Millipore). The membranes were blocked with Tris-buffered saline (TBST) containing 0.1% Tween-20 (Nacalai Tesque Inc.) and 5% Difco Skim Milk (232100, BD Biosciences, Franklin Lakes, NJ) and incubated overnight at 4°C with primary antibodies. Rabbit anti-cleaved caspase-9 antibody (20750S, 1: 1,000) and rabbit anti-*β*-actin antibody (#4967S, 1: 1,000) were obtained from Cell Signaling Technology, Inc. After washing with TBST, the membranes were further incubated with horseradish peroxidase (HRP)- conjugated secondary antibody (1 : 10,000, Santa Cruz Biotechnology Inc.) for 1 h at room temperature and finally developed with an electrochemiluminescence system (ECL) (GE Healthcare, Little Chalfont, UK). The bands were detected using a LAS4000 Mini (Fujifilm, Tokyo, Japan), and the intensities were quantitatively measured by calculating integrated grayscale densities in consistently sized windows incorporating each band using ImageJ software (version 1.48).

### 2.6. Statistical Analysis

Comparisons of data between groups were analyzed using Student's *t*-test. In the case of score values, Mann–Whitney *U* test was used. Fisher's exact test was used for the difference of distribution. A *P* value of less than 0.05 was considered statistically significant.

## 3. Results

### 3.1. Taurine Ameliorates Tumor Load in AOM/DSS Mice

The AOM+DSS mouse model was induced by intraperitoneal injection of AOM followed by two cycles of DSS exposure (Figure 1). One mouse died at week 3 in the model group (*n* = 9 to be *n* = 8) before the termination of the experiment, while no mouse died from taurine group (*n* = 9) and control group (*n* = 3).

**Figure 2:**
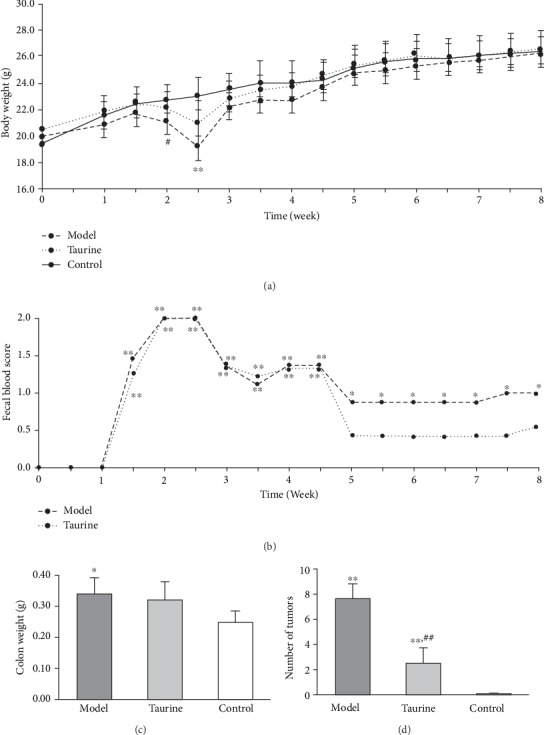
Changes in (a) body weight and (b) fecal blood scores of mice and averages of (c) colon weight and (d) number of tumors. ^∗^*P* < 0.05, ^∗∗^*P* < 0.01 vs. control group, #*P* < 0.05, ##*P* < 0.01 vs. model group by (a, c, d) Student's *t*-test and (b) Mann–Whitney *U* test.

Figure 2(a) shows the body weight change. Mice in the control group gradually gained body weight. Mice receiving DSS lost some body weight during and after the first DSS cycle, and then, the body weight was restored within the interval period. The second DSS also affected the body weight, but lesser than the effect in the first exposure. Mice in the taurine group showed less body weight loss than those in the model group.

Mice in the control group had no fecal blood during the experiment (score = 0 at all time points). All mice receiving DSS showed gross bleeding (score = 2) in feces at the end of the first cycle (week 2, Figure 2(b)). Then, mice were randomly divided into two groups (model group and taurine group). The fecal blood score decreased during the interval period and then slightly increased during the second exposure of DSS. After two cycles of DSS, the mean scores decreased until week 5 and later plateaued (score 1 in the model group and 0.5 in the taurine group). The model group showed significantly higher fecal blood scores than the control group (*P* < 0.05 at least) during and after DSS treatment until the sacrifice. In contrast, the taurine group exhibited no significant differences after week 5. Colon weight (Figure 2(c)) was significantly greater in the model group than in the control group. There was no significant difference between the taurine and control groups, and also between the taurine and model groups. The mean number of tumors (standard deviation, SD) was 7.6 (1.2) in the model group and 2.4 (1.3) in the taurine group (Figure 2(d)). No tumor was observed in the control group. Taurine significantly suppressed AOM+DSS-induced tumor formation (*P* < 0.01). The treatment of AOM, a mutagenic agent, and DSS-induced inflammation for the mouse model of colon cancer is valuable in the understanding of the mechanisms of inflammation in tumorigenesis. In DSS treatment, colitis occurred as observed in the data of gross/occult bleeding and body weight loss. As shown here, taurine alleviated these outcomes.

### 3.2. Taurine Attenuates AOM-DSS-Induced Colon Carcinogenesis

**Figure 3:**
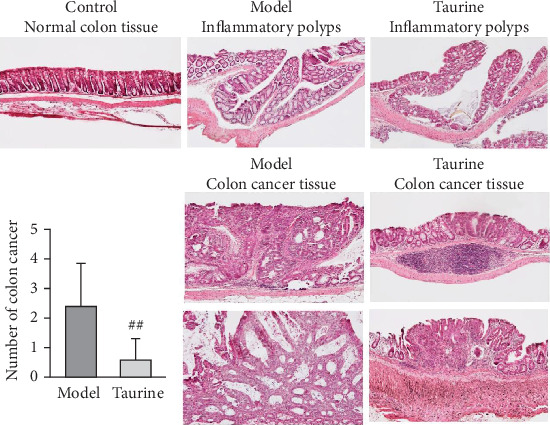
Microscopic examination of colon tissues using H&E staining. Representative images of the normal tissue from the control group, and inflammatory polyps and colon cancer from model and taurine groups. Magnification; 100–200x. Graph represents the average number of colon cancer per mouse (bar; SD). ##*P* < 0.01 between the model and taurine groups by Student's *t*-test.

H&E staining (Figure 3) showed that no inflammation and cancer lesion were observed in the control group. Many inflammatory polyps were observed in both model and taurine groups, but cancer lesions in the taurine group were smaller than those in the model group. Microscopic examination revealed that AOM/DSS treatment induced colon cancer in all mice (8/8, 100%), and taurine inhibited the progression of colon cancer (4/9, 44.4%, *P* < 0.05 by Fisher's exact test). Taurine significantly suppressed the average number of colon cancer compared to that of the model group (*P* < 0.01, Figure 3, graph).

### 3.3. Taurine Inhibits Cell Proliferation and Induces Apoptosis through Activation of PTEN

**Figure 4:**
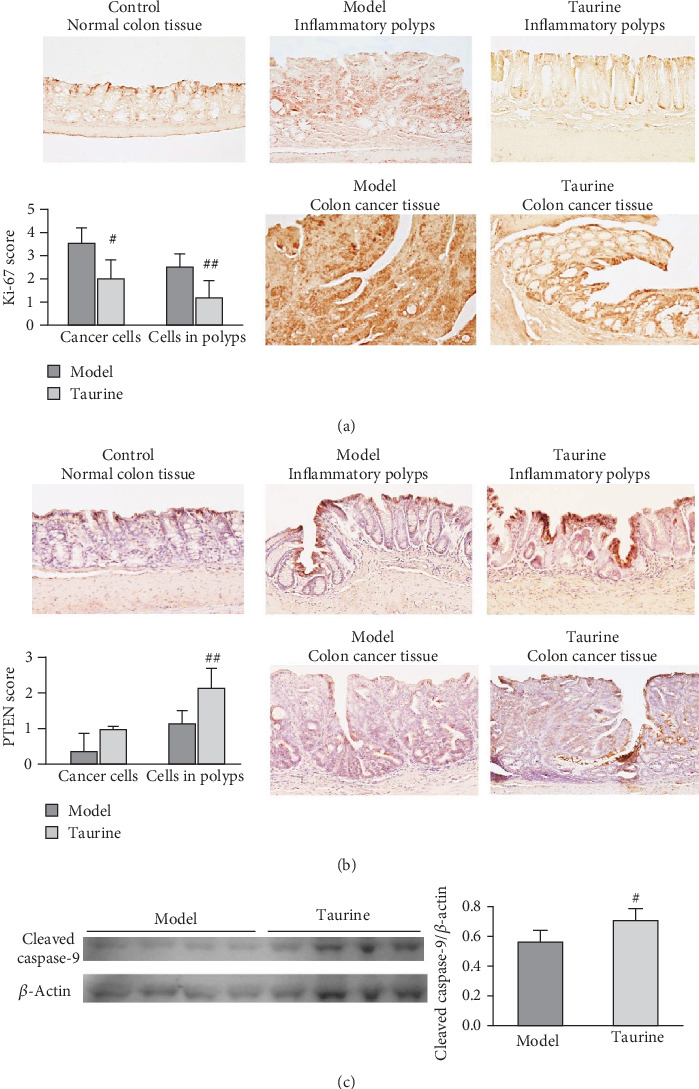
Immunohistochemistry of (a) Ki-67 and (b) PTEN and western blot analysis of (c) cleaved caspase-9. The expression of Ki-67 and PTEN was assessed by avidin-biotin kits with peroxidase-based detection (brown). Nuclei were counterstained with hematoxylin. Original magnifications 100x. (c) Western blot image and relative intensity of cleaved caspase-9 adjusted by *β*-actin. Graphs represent the average score (bar; SD) of (a) Ki-67 score, (b) PTEN score, (c) cleaved caspase-9. #*P* < 0.05, ##*P* < 0.01 between model and taurine groups by Mann–Whitney *U* test for score values and Student's *t*-test for the intensities in the western blot analysis.

Figure 4(a) shows levels of a marker of cell proliferation, Ki-67, in the colon tissues. The intensive Ki-67 immunoreactivities were observed in a large proportion of colon cancer cells in the model group. Ki-67 was expressed in the nuclei of cancer cells and also showed strong immunoreactivities in the epithelial cells adjacent to inflammation polyps in the model group. In the taurine group, the cancer cells showed relatively weak Ki-67 staining in the nuclei. Normal control colon epithelial cells showed a weak immunoreactivity of Ki-67. There were significant decreases in Ki-67 immunoreactivities in both cancer and polyp tissues of taurine-treated mice compared with those of the model mice (Figure 4(a), graph).

Figure 4(b) shows the levels of PTEN, a tumor suppressor. PTEN expression was scarcely detected in the tumor area of colon cancer tissues in the model group, compared to that in the taurine group. The PTEN expression was intensively expressed in the cytoplasm and colon mucosal epithelial cells adjacent to colon cancer tumor area of the taurine group compared to that of the model group. In contrast, normal control epithelial cells showed relatively weak immunoreactivity for PTEN expression. There was a significant increase in PTEN immunoreactivities in polyp tissues of taurine-treated mice, compared with those of model mice (Figure 4(b), graph).

Western blot analysis (Figure 4(c)) showed that taurine increased cleaved caspase-9 level, suggesting taurine-induced apoptosis.

## 4. Discussion

The present study demonstrated that taurine attenuated carcinogenesis in AOM-DSS model mice. At the first time, we had performed a single intraperitoneal injection of AOM at a dose level of 10 mg/kg body weight, and on the 7th day after the injection of AOM, mice received 2% DSS in drinking water for one week, according to the protocol of Suzuki et al. [12]. In our primary experiment, the high mortality of mice was observed after drinking 2% DSS, provably due to the difference of mouse species and age. So, we changed the protocol of 2% DSS into 1% DSS twice with one-week interval. The treatment of AOM and DSS induced colon cancer in all mice (8/8, 100%) of the model group, although one mouse died at week 3. Therefore, it is suggested that our protocol is an adequate method for AOM/DSS-induced mouse model for colon carcinogenesis. Interestingly, we found that taurine reduced the number of colon cancer with lower fecal blood score. Zhang et al. [14] showed antitumor properties of taurine to inhibit cell proliferation and induce apoptosis in colorectal cancer cells *in vitro*. The present *in vivo* study indicates that taurine exhibits an anticancer effect in ulcerative colitis-colorectal cancer mouse model.

In the present study, we observed the increase in cleaved caspase-9 level and decrease of Ki-67 level in mouse colon tissues of the taurine group compared to those of AOM-DSS model group. Takano et al. indicated that apoptosis in colon cancer is related to proliferative activity that can be assessed using Ki-67 labeling [15]. Moreover, several studies showed that taurine induced cell apoptosis in the colon [14], lung [16], and breast cancer cells [17]. This study demonstrated that taurine can significantly enhance the cleaved form of caspase-9, suggesting that the mitochondrial pathway of apoptosis [18] is involved in taurine-induced apoptosis in colon cancer. Our previous *in vitro* studies demonstrated that taurine increased the PTEN level in human nasopharyngeal carcinoma cells, as its anticancer mechanism [10, 11]. PTEN regulates cell division and apoptosis and helps to prevent uncontrolled cell growth, which can suppress tumor formation. PTEN interacts with p53 and enhances p53 stability, resulting in cell cycle arrest and apoptosis [19]. The present study demonstrated that PTEN increased in mouse colon tissues of the taurine group compared to that of the AOM-DSS model group. Taurine-induced PTEN may function as a tumor suppressor, leading to reduction in colon cancer in the mouse model.

Inflammation promotes various pathogeneses, including cancer [20]. Marcinkiewicz and Kontny [2] reviewed a possible contribution of taurine to protect against the pathogenesis of inflammatory diseases. Sun et al. [21] showed that taurine suppressed the inflammatory reaction related to NF-*κ*B in ischemic rat brain damage. Our results suggested that taurine reduced DSS-induced inflammation as observed in lower fecal blood scores. In addition to its anti-inflammation activity, PTEN activation is one of the anticancer mechanisms of taurine.

## 5. Conclusions

This is the first study that demonstrated that taurine significantly reduced carcinogenicity *in vivo* using AOM/DSS-induced colon cancer mouse model. It is suggested that taurine attenuates cell proliferation and induces apoptosis via PTEN induction. Taurine could contribute to the suppression of inflammation-related carcinogenesis.

## Data Availability

All data are available on request to the corresponding author.
